# Optimization of a tannase-assisted process for obtaining teas rich in theaflavins from *Camelia sinensis* leaves

**DOI:** 10.1016/j.fochx.2022.100203

**Published:** 2022-01-03

**Authors:** Shuang Liang, Fang Wang, Jianxin Chen, Daniel Granato, Lijun Li, Jun-Feng Yin, Yong-Quan Xu

**Affiliations:** a Tea Research Institute Chinese Academy of Agricultural Sciences, Key Laboratory of Tea Biology and Resources Utilization, Ministry of Agriculture, 9 South Meiling Road, Hangzhou 310008, China; b Graduate School of Chinese Academy of Agricultural Sciences, Beijing 100081, China; c Department of Biological Sciences, Faculty of Science and Engineering, University of Limerick, V94 T9PX Limerick, Ireland; d College of Food and Biological Engineering, Jimei University, Xiamen 361021, China

**Keywords:** Liquid fermentation, Tannase, Theaflavins, *Camellia sinensis*, Tea leaves, Epigallocatechin gallate, Functional beverages, (−)-Epigallocatechin gallate (PubChem CID65064), (−)-Epicatechin gallate (PubChem CID367141), (−)-Epigallocatechin (PubChem CID72277), (−)-Epicatechin (PubChem CID72276), (−)-Gallocatechin gallate (PubChem CID199472), (−)-Catechin gallate (PubChem CID6419835), (−)-Gallocatechin (PubChem CID9882981), (+)-Catechin (PubChem CID1203), Gallic acid (PubChem CID370), (−)-Theaflavin (PubChem CID135403798), Theaflavin 3-gallate (PubChem CID136825044), Theaflavin 3′-O-gallate (PubChem CID71307578), Theaflavin 3,3′-di-O-gallate (PubChem CID135403795)

## Abstract

This work aimed at optimizing the extraction of theaflavins for the development of a potentially functional tea beverage using different technological parameters as factors. Green tea leaves treated with tannase provided a beverage with significant higher amount (4.7-fold) of theaflavin (TF) compared to the pure withered leaf fermentation. For black tea, the optimized process conditions to produce a beverage with high TF (0.269 μg/mL) concentration were: 6 g of leaves/400 mL, a low fermentation temperature of 25 °C with the absence of buffer and pH control, an intermediate fermentation time (60 min) and a relatively low aeration rate (0.8–1.0 L/min). The tea liquid produced under optimized fermentation conditions appears to be ideal for making a black tea beverage with surplus summer tea leaves and brings economic benefits.

## Highlights

• Oxidation with tannase-treated green tea extract produced higher TFs content juice.
• Hydrolyzing ester catechin increased TFs content in the liquor by up to 4.7-fold.
• Optimized preparation parameters are 6 g, 25 °C, 60 min and 0.8–1.0 L/min.
• Preparation of high TFs black tea juice from low-end fresh tea leaves.

## Introduction

Tea is one of the most consumed non-alcoholic beverages in the world, and black tea is the most consumed tea product, accounting for about 75% of global tea consumption (Zhang, Ho, et al., 2019). Liquor brightness and total color of black tea are critical quality attributes, used in the tea trade to rank and price black teas, and these attributes depend primarily on the content of theaflavins (TFs) and thearubigins (TRs) (Dutta, Stein, & Bhagat, 2011). The TFs content of black tea processed by traditional Chinese methods is generally around 0.5%, while the TFs content in the black tea produced in India, Sri Lanka and other countries is about 2%, higher than those in Chinese black tea (Ye et al., 2015, Ma et al., 2016). The flavor of Chinese black tea is not thick, strong and fresh with the qualities sought in the international market, thus Chinese black tea has a very small market share. However, according to the Food and Agriculture Organization of the United Nations, China is the largest planting and producing country of tea leaves. To improve the international market share of Chinese black tea, new processes must be developed and implemented to increase the TFs content and improve the quality of black tea (Ma, Cheng, & Tu, 2016).

It is still hypothesized that teas made from fermented leaves suspended in water could increase the concentrations of TFs and TRs, and produce a better-quality black tea beverage (Zhou, Chen, Huang, Fu, & Ni, 2013). In the 1980 s, purified green tea catechins and partially purified polyphenol oxidase (PPO) (EC1.14.18.1) in a simulated fermentation system have been used to increase the content of TFs and TRs through *in vitro* simulation of industrial fermentation (Robertson, 1983). Compared with the traditional black tea fermentation process, crushing the fresh leaves before liquid fermentation would improve the access of the PPO (whether endogenous to the leaves, or exogenous) to its substrates. Liquid fermentation requires a shorter time and gives a more extensive oxidation reaction (Yang, 2011), achieving a reliable fermentation and producing high quality black tea (Xia et al., 2000, Shen et al., 2019), with a high TFs content. In addition, TFs have multiple medical and health benefits, such as antioxidant (Tan et al, 2018), anticancer (Xu, Zhao, Cao, Tang, Gan, & Li, 2019), antimicrobial (Hui, Yue, Zhang, Li, Yang, & Gao, 2016), anti-inflammatory (Xu, Zheng, Tang, Liu, Hu, & Shang, 2021) and antiviral effects (Mhatre, Srivastava, Naik, & Patravale, 2020). In addition, TFs extracted from black tea have higher DPPH free radical scavenging and hydrogen peroxide quenching capacity than a TRs extract (Ali, Muhammad, Muhammad, Muhammad, Farhan, & Muhammad, 2018).

There are many factors influencing the TFs content of liquid-fermented tea. The ratio of PPO activity to peroxidase (POD) activity in different tea varieties significantly impact on the production of TFs (Jiang, Hua, Yuan, & Ma, 2018), as does the catechins composition (Yang et al., 2011, Hua et al., 2020). The addition of exogenous catalase to a liquid fermentation of low-grade green tea produced more like black tea pigments by synergy with PPO compared to PPO alone (Zhong, Zheng, Zeng, & Deng, 2016). Since TFs can be oxidized to form TRs in a liquid fermentation, increasing the TFs content in black tea requires inhibition of the transformation of catechins and TFs to TRs. Experimental evidences have shown that TFs and TRs contents are positively correlated with fermentation time (Muthumani & Kumar, 2007) and decreasing fermentation temperature could increase TFs production and inhibited TRs production (Robertson, 1983). Extractions at pH 4.5–4.8 increase the TFs content, and decrease the TRs content and improve the sensory attributes of the final product (Cloughley, & Ellis, 1980). Additionally, increasing the oxygen flux by aeration activates PPO and thereby increases the TFs production (Robertson, 1983, Xia et al., 2000). The optimal process parameters for fresh leaf liquid fermentation conditions were obtained by response surface methodology (RSM): fermentation temperature, 35.3 °C; fermentation time, 60.3 min; oxygen flux, 1.04 L/min. These conditions maximized the TFs and TRs content and TFs/TRs ratio (13.05), meeting the quality requirements for black tea liquor color (Shen, 2009).

Some studies aimed at optimizing the process parameters of liquid suspension fermentation, usually by a single factor experiment, combined with orthogonal design (Shen, Xu, Si, Pan, Zhang, & Zhao, 2019) or RSM (Shen, 2009, Zhou et al., 2013). There have been few studies on the use of tannase (EC 3.1.1.20; catalyzes hydrolysis of galloyl ester bonds in gallotannins) to hydrolyze gallotannins in combination with a liquid fermentation system. Considering this technological need and scientific gap in tea production, this study aimed to incorporate tannase in tea leave fermentation process in order to increase the TFs content of liquid-fermented black tea juice (LFBTJ)**.**

## Materials and methods

### Materials and reagents

Fresh leaves of Longjing 43 tea plants were plucked according to the standard method of one bud and four leaves, and then immediately stored frozen at −20 °C. Edible defoamer for bean products was from a food additives company in Hebei Province, China. Green tea pieces (Longjing) were from Hangzhou Longguan Industrial Co., Ltd. (China). Tannase (5000 U/g) was from Heilongjiang Weinuoen Biotechnology Co., Ltd. (China). Gallic acid (GA), gallocatechin (GC), epigallocatechin (EGC), catechin (C), epicatechin (EC), gallocatechin gallate (GCG), epigallocatechin gallate (EGCG), catechin gallate (CG), epicatechin gallate (ECG), TF, theaflavin-3-gallate (TF-3-G), theaflavin-3′-gallate (TF-3′-G) and theaflavin-3–3′-digallate (TFDG) were from Shanghai YuanYe Biotechnology Co., Ltd. (China) with purity of ≥ 95%. Citric acid monohydrate, disodium hydrogen phosphate (analytical grade), acetonitrile, acetic acid, water (chromatography grade) and other chemicals were from local suppliers. Purified water was from Hangzhou Wahaha Beverage Co., Ltd. (China).

### Fermentation using different tannase treatment methods

**Method 1** involved fermentation of fresh tea leaf homogenate with added tannase. Frozen fresh tea leaves were withered at room temperature (22 ± 1 °C) for 1 h, and weighed 12 g for each sample. The samples (12 g) with purified water (200 mL) were homogenized for 2 min (2nd gear, 30 s each time, 4 times) in a 4186 Food Processor (Braun Co., Ltd., Czech Republic). The homogenate was transferred to a beaker in a 35 °C water bath, then defoamer (0.8 mL) and purified water (200 mL) were added. The fermentation medium (total volume 400 mL) was aerated at 1.0 L/min (ACO-003 air pump, RESUN Group Co., Ltd., China), then 5 U/mL tannase solution (0.5 mL to 2.5 U, 1 mL to 5 U, or 2 mL to 10 U) was added to the mixture. Every 10 min, a 5 mL aliquot was removed and the enzyme inactivated in an X3-233A microwave (Midea Group, China) for 10 s (heating to 90 °C), then cooled to room temperature. The above treatment was conducted in triplicate.

**Fig. 1:**
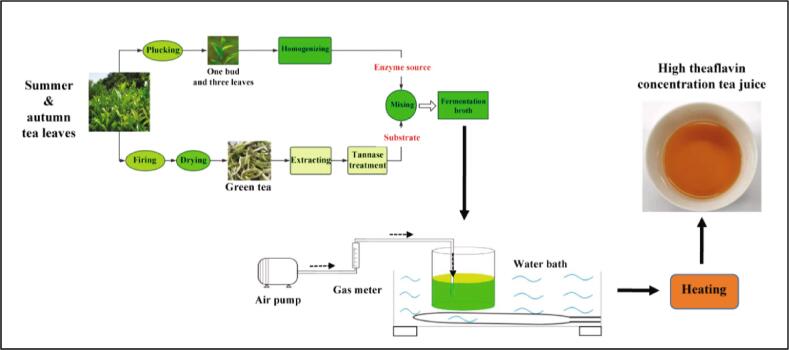
Technical route for preparing high theaflavin content black tea juice from summer and autumn tea.

**Method 2** involved fermentation of fresh leaf homogenate with tea broth, previously hydrolyzed by tannase (Fig. 1). Longjing green tea leaves (10 g) were added to boiling water (450 mL), maintained at 100 °C in a water bath for 20 min, and then filtered hot with a 500-mesh filter cloth, the filtrates were diluted to 500 mL, and then cooled to room temperature. The resulted green tea extract (200 mL) was treated with 0.05% or 0.1% w/w tannase and maintained at 50 °C in a water bath for 25 min, heated to 90 °C with the microwave, then cooled to room temperature.

Frozen fresh leaves were withered, homogenized with water, or buffer and transferred to the fermentation beaker as for Method 1. Defoamer (0.8 mL) and the tannase-treated extract (above; 200 mL; substituted for the purified water) were added, then the fermentation performed as for Method 1, except that 2 mL samples were taken. The above treatment was conducted in triplicate.

### Single factor experiments

Preparation of green tea extract, tannase hydrolysate of green tea extract and LFBTJ was as in **Method 2** in Section 2.2, but with each of the following factors were varied, while keeping the others constant, i.e.: quantity of withered leaves (14.0, 12.0, 10.0, 8.0, 6.0, or 4.0 g), fermentation medium (pure water, or citric acid-phosphate buffer at a pH of 4.0, 4.5, 5.0, 5.5, 6.0, or 6.5), fermentation temperature (25, 30, 35, 40, 45, or 50 °C), fermentation time (30, 45, 60, 75, 90, or 105 min) and aeration rate (0.6, 0.8, 1.0, 1.2, 1.4, or 1.6 L/min). All experiments results were from three separate experiments.

### Evaluation of total soluble solid content and color

The total soluble solid content of fermented black tea juice was determined using an RX-0007CX refractometer (Atago Co., Ltd.). Chromatic analysis (using CIELAB color space - L*a*b*) of black tea juice (diluted 5 times) was performed with a Minolta CT-310 color analyzer (Konica-Minolta (China) Investment Ltd.).

### Determination of catechins by high performance liquid chromatography (HPLC)

Catechins were determined by HPLC, as described previously (Wang, Yang, Wang, Zeng, Xu, & Yin, 2020). The samples of tea infusion were filtered through a 0.45 μm Millipore filter before injection. The detection conditions were as follows: column with 5 μm Diamonsil™ C18 (4.6 mm × 250 mm; Dikma Technologies Inc., Lake Forest, CA); temperature at 35 °C; post-run time for 5 min; injection volume was 10 μL; the flow rate was 1.0 mg/mL; detection wavelength at 280 nm. The mobile phase comprised A: 2% v/v acetic acid; mobile phase B: 100% acetonitrile. The elution gradient was as follows: 0–16 min, 6.5% B; 16–25 min, 15% B; and 25–30 min, 6.5% B. The standard catechins, GA and caffeine (GC: y = 1*e^6^x, R^2^ = 0.9994; EGC: y = 2*e^6^ ×, R^2^ = 0.9995; C: y = 7*e^6^ ×, R^2^ = 0.9991; EC: y = 8*e^6^x, R^2^ = 0.9998; GCG: y = 2*e^7^x, R^2^ = 0.9997; EGCG: y = 1*e^7^x, R^2^ = 0.9998; CG: y = 2*e^7^x, R^2^ = 0.9990; ECG: y = 2*e^7^x, R^2^ = 0.9999; GA: y = 2*e^7^x, R^2^ = 0.9993; caffeine: y = 3*e^7^x, R^2^ = 0.9993) were used for identification and quantitative analysis.

### Determination of TFs by HPLC

Theaflavins were determined in triplicate by HPLC (LC-20A HPLC, Shimazu (China) Co., Ltd.) employed with a Symmetry C18 column (Waters Corporation, Milford, MA) with a flow rate of 1.5 mL/min, column temperature of 35 °C, injection volume of 10 μL and detection at 280 nm. The mobile phases were (A) 2% acetic acid and (B) acetonitrile. The mobile phase composition was 20% B from 0 to 12 min, then 22% B at 12.5 min, maintained at 22% B until 16.5 min, ramped linearly to 20% B at 17 min, maintained until 20 min. The standard theaflavins (TF: y = 1*e^7^ ×, R^2^ = 0.9999; TF-3-G : y = 7*e^6^ ×, R^2^ = 0.9998; TF-3′-G: y = 1*e^7^x, R^2^ = 1; TFDG: y = 4*e^6^x, R^2^ = 0.9999) were used for identification and quantitative analysis.

### Statistical analysis

All experimental data are presented as the mean ± standard deviation of three replicates. The biochemical composition map of each treatment was plotted using GraphPad Prism 8 (GraphPad Software, San Diego, CA). Response surface plots were generated using Design-Expert 8.0.6 (Stat-Ease Inc., Minneapolis, MN). Data analysis was performed using IBM SPSS Statistics 25 (SPSS Inc., Chicago, IL), and significant differences between samples were analyzed by ANOVA and Duncan’s multiple range test. *P* < 0.05 was considered statistically significant.

## Results and discussion

### Modulation of liquid-fermentation and TFs formation using different tannase treatments

#### Withered leaf homogenate with exogenous tannase (**Method 1**)

**Fig. 2:**
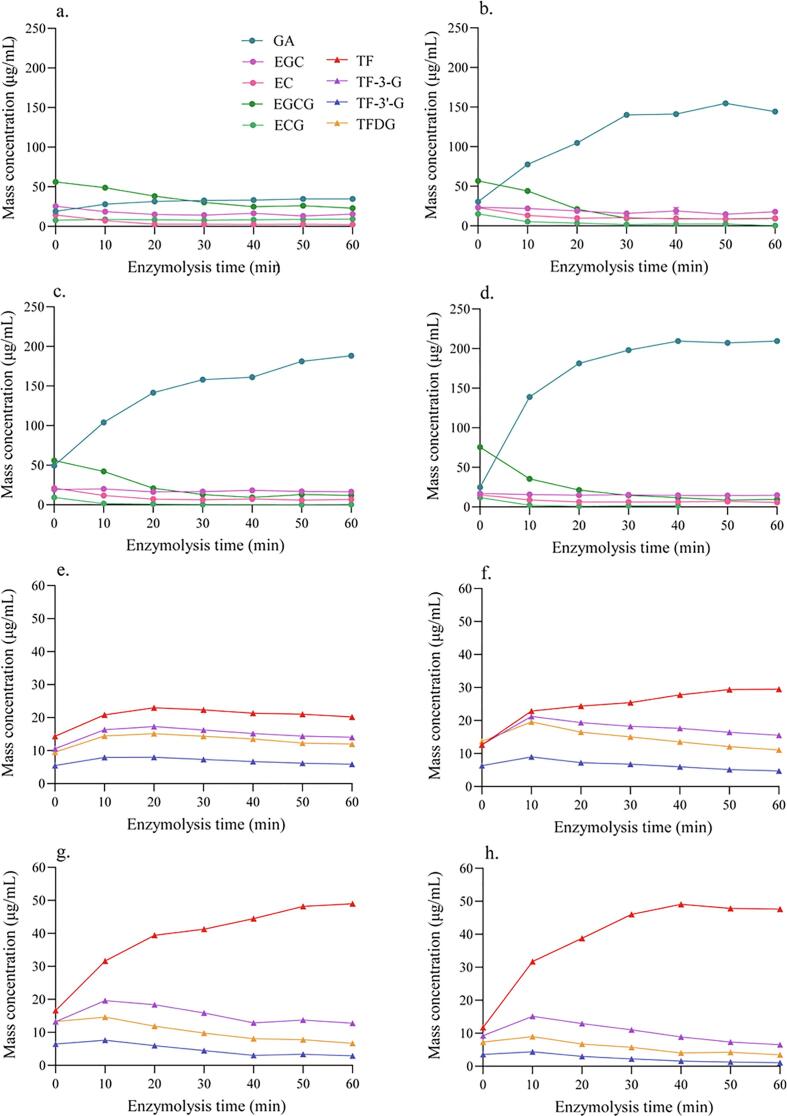
**Effects of different amounts of exogenous tannase in mixed fermentation of withered leaf homogenate on the concentrations of catechins (a-d) and theaflavins (TFs, e-h)** (a, e) no tannase control (CK), (b, f) 2.5 U tannase, (c, g) 5 U, and (d, h) 10 U. Note: TF-3-G (TF-3-gallate); TF-3-G (TF-3-gallate); TFDG (TF-3,3-digallate).

The effects of enzymatic fermentation time on the catechin concentrations during fermentation are determined (Fig. 2). In the control samples (CK, Fig. 2a), the EGCG concentration was decreased from up to 40 min, and then plateaued, whereas the GA concentration increased up to 32 μg/mL at 30 min, and then plateaued. This observation may be attributed to enzymes that hydrolyze catechin esters in fresh tea leaves, such as endogenous tannase, which accounts for the hydrolysis of EGCG to GA and EGC. Exogenous tannase significantly accelerated catechin ester hydrolysis, decreasing EGCG and increasing GA in a dose-dependent manner (Fig. 2b ∼ d). The production of GA reached 150 μg/mL at 50 min, then declined slightly with 2.5 U of tannase. With 5 U of tannase, the GA concentration increased up to 60 min and reached to 188 μg/mL (Fig. 2b). Using 10 U of tannase, the GA concentration reached to 209 μg/mL (6.5-fold that in CK) at 40 min, then plateaued (Fig. 2d). The effect of endogenous enzymes was negligible, whereas even 2.5 U of exogenous tannase increased 5-fold the GA concentration.

The TF concentration reached 20 μg/mL at 60 min in CK (Fig. 2e), which followed the method used in Shen’s experiments (Shen, 2009). Using 2.5 U exogenous tannase (Fig. 2f), TF reached to 29.5 μg/mL at 50 min and then plateaued. When 5 U tannase (Fig. 2g) was employed, TF reached to 49 μg/mL (2.1-fold that in CK) at 60 min. Similarly, when 10 U tannase (Fig. 2h) was used, TF reached 47.6 μg/mL at 60 min. While TF-3-G, TF-3′-G, and TFDG increased at 10 min under all four conditions, then decreased in a dose-dependent manner when exogenous tannase was added in the beverage model system. It is probable that part of the increase in TF is associated with the hydrolysis of TF-3-G, TF-3′-G, and TFDG. The results indicated that the total concentration of theaflavins was maximized when 5 U tannase (71 μg/mL, ∼1.4-fold that in CK) was used. Those results were consistent with TF formation catalyzed by endogenous PPO (Yang, 2016). Fermentation of autumn withered tea leaf homogenate with exogenous tannase (5 U, the optimal concentration) employed in **Method 1** significantly decreased EGCG and increasing GA in a dose-dependent manner, and increased the TF concentration of LFBTJ (2.1-fold that in CK), higher than previous studies (Shen, 2009). However, the contribution of tannase to the hydrolysis of TF-3-G, TF-3′-G, and TFDG cannot be easily determined. Therefore, the tannase treatment and fermentation processes were performed as separate steps, using **Method 2**.

#### Withered leaf homogenate mixed fermentation with tannase-hydrolyzed green tea extract (**Method 2**)

To compare the individual effect of exogenous tannase on TFs formation with those of endogenous tea leaf enzymes, green tea extract was treated with tannase, then subsequently added to the leaf homogenate fermentation, thereby increasing the TFs and catechins contents. The addition of 0.05% tannase had limited effect on the composition of green tea extract after 2 h (supplementary Fig. S1a), whereas 0.25% tannase completed the hydrolysis within 15 min (supplementary Fig. S1b). However, 0.1% tannase was able to complete the hydrolysis within 30 min, producing a moderate rate of reaction, neither fast nor slow (supplementary Fig. S1c), so 0.1% tannase was chosen as the optimal concentration to use to be implemented in **Method 2**.

**Fig. 3:**
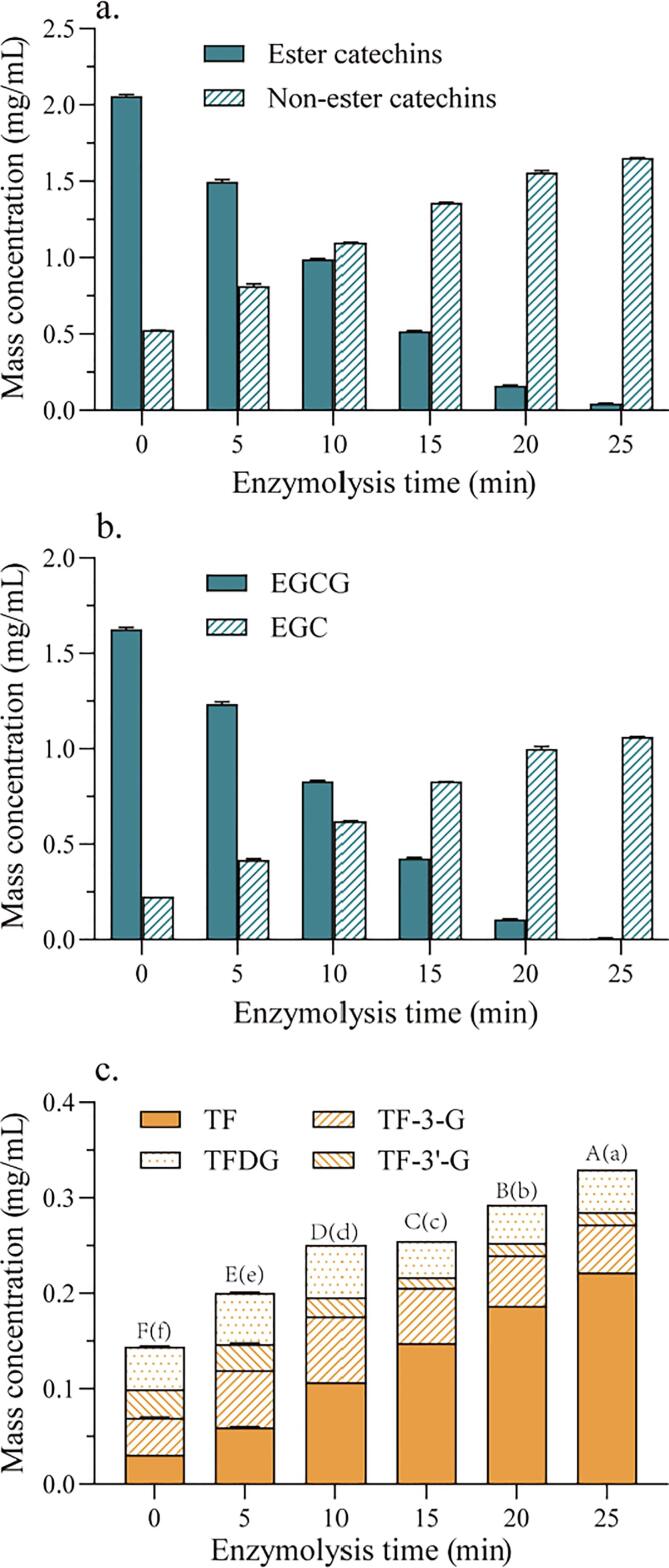
**Effects of 0.1% w/w tannase on green tea extract.** (a) esterified and non-esterified catechin content; (b) EGCG and EGC content; (c) content and composition of TFs in black tea juice made by Method 2, after mixing with green tea extract hydrolyzed by 0.1% w/w tannase for 0, 5, 10, 15, 20, or 25 min. (For interpretation of the references to color in this figure legend, the reader is referred to the web version of this article.)

The treatment of green tea extract with 0.1% tannase was repeated over a shorter time to obtain more detailed time-courses (Fig. 3). After 25 min, most of the ester catechins were hydrolyzed (Fig. 3a), as well as all of the EGCG (Fig. 3b). There was a significant positive correlation (r = 0.983, *P* < 0.01) between the ratios of non-ester/ester catechins and of EGC/EGCG, so that the EGC/EGCG ratio can be considered as a good indicator of the degree of hydrolysis of total ester catechins.

Black tea beverage was prepared by fermentation of withered leaf homogenates after mixing with green tea extract pre-hydrolyzed by 0.1% w/w tannase for 0, 5, 10, 15, 20, or 25 min (Fig. 3c). The TF concentration ranged from 31 (no tannase hydrolysis) to 222 μg/mL (25 min hydrolysis). The lowest TF concentration was found from the negative control treatment (no tannase). The results showed noticeable better quality with high TF concentration in the treated tea with tannase-treated green tea extract compared to the samples obtained from **Method 1** (processing without exogenous tannase treatment (Fig. 2a). The treatment of the green tea with tannase for 5 min, the TF concentration reached 59 μg/mL, which is virtually higher (P < 0.05) than the highest value obtained with **Method 1** (59 μg/mL). Green tea treated with tannase for 25 min reached a TF content of 220 μg/mL, about 4.7 fold that from **Method 1**. After 25 min of tannase treatment, total TFs reached 330 μg/mL, about 2.4-fold compared to the control treatment (without tannase). Therefore, **Method 2** is clearly superior compared to Method 1 in providing a beverage with higher TF concentration. It is clearly advantageous to add tannase-treated green tea extract to black tea fermentations, because it provides more material for TF formation. This approach also seems to be unaffected by substrate inhibition as observed previously (Xia et al., 2000).

The TF composition of liquid-fermented black tea juice was dependent on the pre-hydrolysis time and the degree of hydrolysis of GA ester catechins reached. The TF-3-G and TFDG concentrations in the treated samples were highest after hydrolysis for 10 min when the degree of hydrolysis was<50% (Fig. 3), whereas the TF-3′-G concentration was higher without pre-hydrolysis, indicating that the TF composition is directly related to the degree of hydrolysis of ester catechins.

### Optimization of TF formation in liquid-state fermentation by using Method 2 with changing the independent variables

#### The amount of withered leaves

Withered leaves (14.0, 12.0, 10.0, 8.0, 6.0, or 4.0 g, in a 400 mL fermentation) were used to manufacture black tea beverage combined with tannase-treated green tea extract. The TF concentrations were increased and reached to highest of TF when the amounts of leavers increased from 4 g to 6 g, and after that TF concentrations decreased with increasing amount of withered tea leaves from 6 to 14 g. (Fig. 3a), whereas the concentrations of TF-3-G, TF-3′-G, and TFDG gradually decreased when the amount of tea leaves increased.

As expected, when the amounts of leaves increased, the total soluble solids concentration of LFBTJ and the values of a* and b* increased (Fig. S2a ∼ c), while the value of L* decreased significantly (Fig. S2d). Increasing amounts of leaves modified the color of black tea beverage, decreasing the brightness and increasing the redness and yellowness. The TF concentration had a strong positive correlation with the L* value (r = 0.902, *P* < 0.01), i.e., decreasing TF concentration increased the brightness of tea juice, but a negative correlation with soluble solids (r = -0.935, *P* < 0.01), a* value (r = -0.895, *P* < 0.01) and b* value (r = -0.806, *P* < 0.01). The redness and yellowness were strongly affected by the solid concentration (r = 0.972 and 0.891, *P* < 0.01).

#### Fermentation temperature

**Fig. 4:**
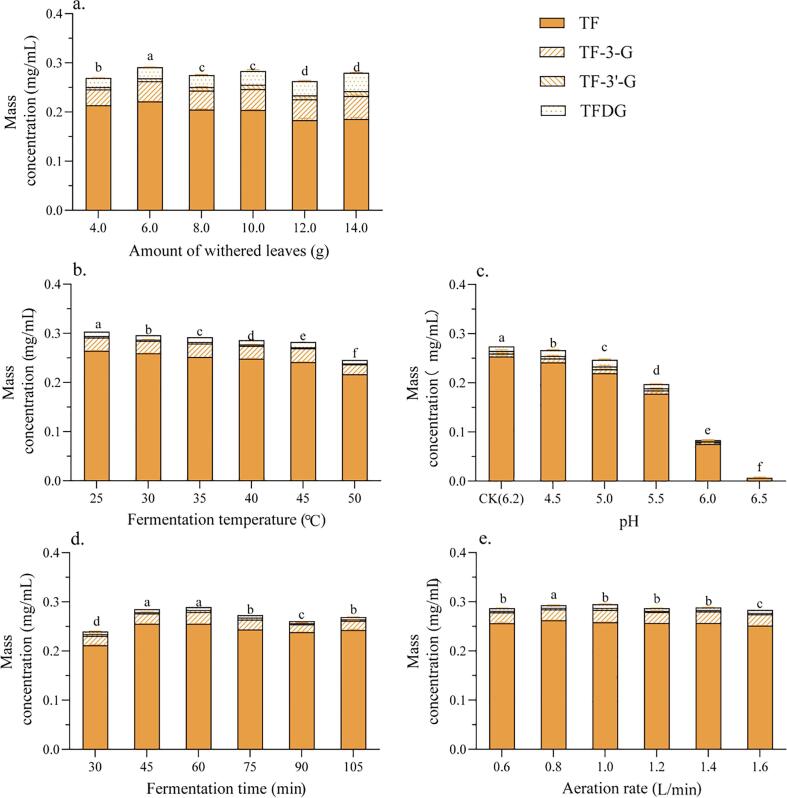
**Effect of independent variables on theaflavin (TF) formation during liquid-state tea fermentation.** (a) amount of withered leaves; (b) fermentation temperature; (c) pH of fermentation medium; (d) fermentation time; (e) aeration rate. Mean values with different lowercase letters indicate significant differences in TF content, based on the least significant difference (LSD) test (P < 0.05). Error bars represent the average standard deviation from six independent experiments, carried out in triplicate. Different letters for the same analytical method represent statistically different results at p < 0.05, A-F from TFs and a-f from TF.

As the fermentation temperature increased from 25 to 50 °C, the TF concentration decreased. The highest TF concentration (264 μg/mL) was obtained at 25 °C, which significantly higher than that at any higher temperature (Fig. 4b). It can be explained that PPO and peroxidase enzymes in tea leaves are not thermostable and lose activity as the temperature increases (Samanta, Cheeni, Das, Roy, Ghosh, & Mitra, 2015), which would account for the highest TF concentration being at 25 °C.

Fermentation temperature had a minimal effect on the soluble solids concentration of LFBTJ (Fig. S2a). With increasing temperature (Fig. S2b), the L* value increased slightly and the a* value decreased slightly (Fig. S2c & d). The results suggested that increase of temperature resulted in increase of the brightness and decreased the redness. The TF concentration had a weak correlation with total soluble solids (r = 0.419, *P* > 0.05), a* (r = 0.481, *P* > 0.05) and b* values (r = -0.103, *P* > 0.05), but a significant negative correlation with the L* value (r = -0.665, *P* < 0.05). In fact, teas produced at the higher temperatures contained more thearubigin pigments (Robertson, 1983) and TRs were negatively correlated with brightness (Obanda., Owuor, Mang’oka, & Kavoi, 2004).

#### The pH of the fermentation medium

The use of buffer to control the pH of the fermentation medium significantly decreased the concentrations of TF, TF-3-G, TF-3′-G and TFDG (Fig. 4c). In the control sample (CK; no buffer, only purified water), the TF concentration was 253 μg/mL and significantly decreased to a minimum of 7 μg/mL at pH = 6.5, with TF-3-G, TF-3′-G and TFDG undetectable when buffer used to control the pH. Clearly, pH control is detrimental to TFs formation and accumulation, and purified water is the best fermentation medium.

Increasing pH led to decrease the L* (brightness) of LFBTJ (Fig. S2b) and markedly increased soluble solids (Fig. S2a), whereas a* and b* were highest at intermediate pH values (Fig. S2c & d). Total soluble solids had a significant and negative correlation with L* (r = -0.888, *P* < 0.01). The TF concentration negatively correlated with soluble solids (r = -0.677, *P* < 0.05) and positively correlated with L* (r = 0.910, *P* < 0.01), a* (r = 0.464, *P* > 0.05), and b* (r = 0.636, *P* < 0.05). In fact, the increase in soluble solids from the addition of citric acid-phosphate buffer and did not favor the TF production. In addition, citric acid and salts increase the sourness and saltiness of tea beverage (Muthumani, et al., 2007), which may negatively affect its taste and sensory acceptance.

#### Fermentation time

The TF content of LFBTJ varied with fermentation time (Fig. 4d). The TF contents reached the highest values at 45 and 60 min (255 μg/mL), and then slightly decreased throughout the fermentation process. The lowest TF concentration was observed at 30 min of fermentation. The fermentation time influenced the color significantly, L* slightly decreased at longer times (Fig. S2b), whereas a* and b* increased up to 60 min, then plateaued (Fig. S2c & d). The TF concentration positively correlated with a* (r = 0.677, *P* < 0.05) and b* (r = 0.758, *P* < 0.01), and negatively with total soluble solids (r = -0.328, p > 0.05) and L* (r = -0.432, p > 0.05). The variation in L* appears to be related to TR formation, since TFs and TRs were reported to increase with fermentation time, but TF reached a maximum, then decreased (Muthumani, & Kumar, 2007). The variation in a* and b* is closely correlated with and clearly related to, TF formation.

#### Aeration rate

TF concentration decreased with increasing the aeration rate from a maximum of 262 μg/mL at 0.8 L/min, to a minimum of 251 μg/mL at 1.6 L/min (P < 0.05) (Fig. 4e). When the aeration rate was increased, the soluble solids was decreased (Fig. S2a), while no significant effects on L*, a*, and b* were observed (Fig. S2b ∼ d). The correlations between the TF concentration and total soluble solids (r = 0.505, p > 0.05), L* (r = -0.187, p > 0.05), a* (r = 0.075, p > 0.05) and b* (r = -0.152, p > 0.05) were marginal and non-significant. Increased oxygen supply increases the degradation of TFs (Robertson, 1983), thus the optimization of the TF concentration and color, the aeration rate should not exceed 1.0 L/min. The results suggested that the TF concentration of black tea beverage was increased by a relatively low addition of withered leaves (6 g in 400 mL), a low fermentation temperature (25 °C), the absence of buffer and pH control, an intermediate fermentation time (60 min) and a relatively low aeration rate (0.8–1.0 L/min).

The chromatic analysis indicated that increased TF concentration correlated with color changes, such as increased brightness, redness and yellowness. These changes are desirable in a black tea-based beverage, whereas total soluble solids were inversely correlated with these changes. A relatively low amount of withered leaves both increased the TF concentration and decreased soluble solids concentration.

### Optimization of modeled parameters by Response-surface methodology (RSM)

Based on the above single-factor experimental design results, the three factors with significant, non-linear effects on TF concentration - amount of withered leaves (A), fermentation time (B), and aeration rate (C) were selected for optimization (Table S1). Following a three-factor Box-Behnken experimental design, the response surface design and experimental results are shown in Table S1.

After fitting the experimental data to quadratic models, the mass concentration of TF was used as the dependent variable (Y), and the withered leaf addition (A), fermentation time (B), and aeration rate (C) were used as independent variables to establish a quadratic polynomial regression equation (Eq. 1):

Y = 0.27 + 0.003625*A* + 0.00425*B* + 0.002375*C* + 0.0005*AB* − 0.00025*AC* − 0.0015*BC* − 0.024*A^2^* − 0.012*B^2^* − 0.010*C^2^*

The model correlation coefficient, *R^2^*, was 0.9876 (Table S2); the coefficient of variation (*CV*) was 3.13%; the signal-to-noise ratio was 20.913, and so > 4; and *R^2^_Adj_* = 0.9717 and *R^2^_Pred_* = 0.8555 were reasonably consistent. These parameters indicate that the regression equation fits the data well.

**Fig. 5:**
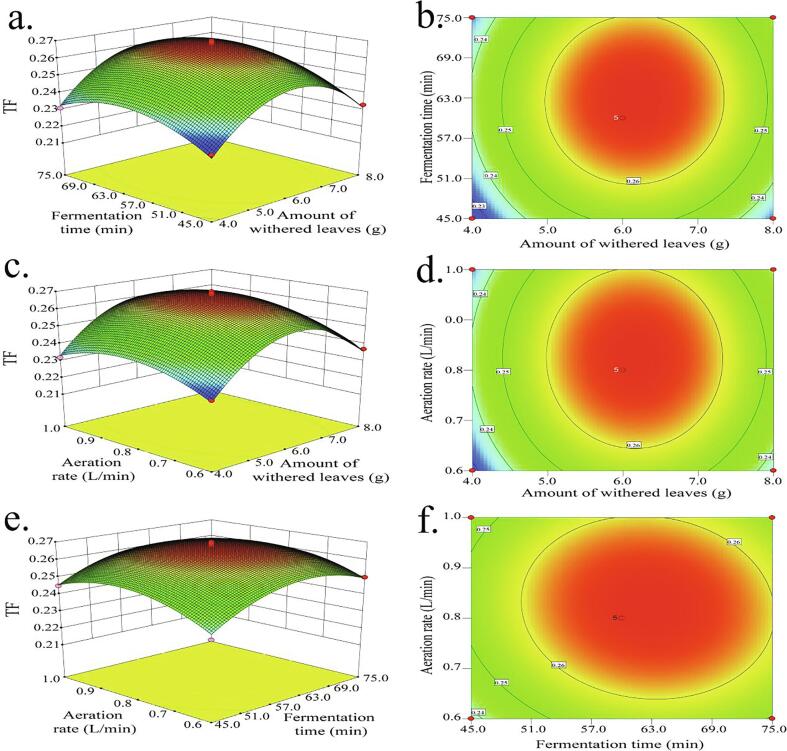
Response surface plots and corresponding contour plots showing the effect of fermentation parameters on the TF concentration.

Response surface plots were to explore the interactions between related variables in depth to determine the optimum values of the three variables (Fig. 5). The variation in TF concentration with the amount of withered leaves (Fig. 5a) and the fermentation time (Fig. 5b) were both increased, and then decreased with the optimum TF concentration obtained with 6.1 g of leaves and a fermentation time of 63 min. In Fig. 5c & d, with the increase withered leaves amount, TF concentration gradually increases, but started to decrease when the amount of tea leaves exceeded 6.1 g. Similarly, when the aeration rate exceeded 0.8 L/min and the TF concentration started decline. As shown in Fig. 5e & f, as the fermentation time increased, TF concentration gradually increased, but when the fermentation time exceeded 63 min, TF concentration decreased. At the same time, when the aeration rate was 0.82 L/min, TF concentration reached its maximum value. It can be seen from the Fig. 5e & f that the contour map was elliptical, indicating that the interaction of various factors was significant.

The optimal liquid fermentation process conditions were predicted by solving the fitting equation (Table 1). To facilitate the operation, the predicted optimal process conditions were revised. After three repeated verification experiments, the average TF concentration was 270 μg/mL, not significantly difference was found from the predicted value of 269 μg/mL thus indicating that the model can be used for prediction purposes.

**Table 1:** The optimal process conditions and validation experiment.

Fermentation parameters	Withered leaf addition amount / (g)	Fermentation time / (min)	Aeration rate / (L/min)	Mass concentration of TF / (mg/mL)
Predicted	6.16	62.63	0.82	0.269 ± 0.000^a^
Revised	6.16	62.66	0.82	0.270 ± 0.001^a^

Note: same latter indicates no significant difference between the groups (*P* < 0.05).

**Fig. 6:**
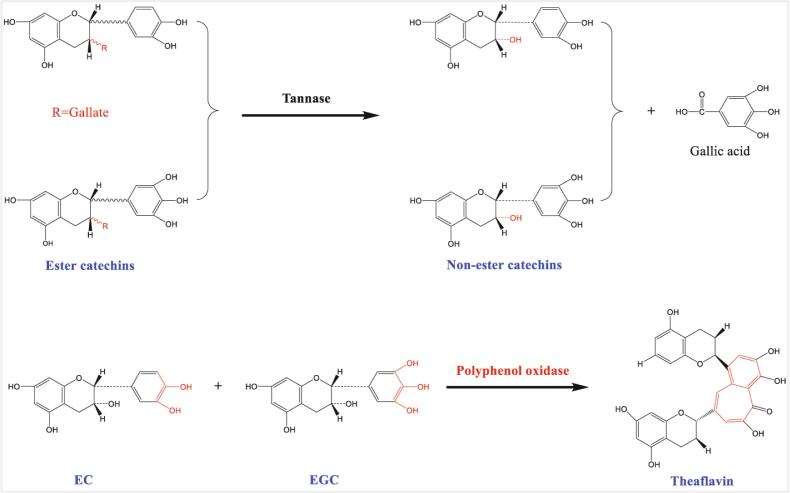
Proposed reaction scheme to explain the increased theaflavin content in liquid-fermented black tea juice, resulting from addition of tannase-treated green tea extract. (For interpretation of the references to color in this figure legend, the reader is referred to the web version of this article.)

Previous studies have investigated the influence of catechin profile and content on the formation of TFs during fermentation of homogenized fresh tea leaf suspensions (Tu, 2014, Jiang et al., 2018, Hua et al., 2020). An EGC/EGCG ratio of 1: 2 resulted in formation of relatively high amounts of TF-3ʹ-G and TFDG, and a significantly higher TFs content. Batchwise addition of catechin substrates for PPO, with a high EGCG content increased TF formation (Hua et al., 2020). Herein, the results showed that the catechins came from green tea extract and the catechin composition was modified by tannase treatment. A longer tannase treatment time produced an EGC/EGCG ratio of 1:130, and meanwhile increased the TF concentration (Fig. 2c) and the TF concentration was proportional to the non-ester/ester catechin ratio. Based on these findings, we propose a mechanism to explain the action of tannase in increasing the TF yield in tea fermentations (Fig. 6).

In China there are over 3.05 million hectares of tea garden, producing 2.609 million tons of tea. Consumer’s demand for summer- and autumn-harvested teas is very low compared to spring-harvested tea, because of their greater bitterness and astringency (Xu, Song, Li, & Wan, 2012). This results in low sales and considerable wastage of summer and autumn tea, because a great portion of harvested tea cannot be marketed and are usually discarded. Autumn tea contains high levels of catechins, especially gallated catechins (mainly EGCG and ECG), which enhance the bitter and astringent tastes (Chen, Jiang, Duan, Shi, Xue, & Yukio, 2009). Thus, technological options aiming to reduce the bitterness and astringency and improve the taste of autumn tea are highly desirable (Cao et al., 2019). Summer and autumn tea would be excellent raw materials for processing with tannase using **Method 2**, because of their high catechin content and ready availability.

## Conclusions

Liquid fermentation of withered autumn tea leaf homogenate with addition of tannase-treated green tea extract produced a black tea liquor with a remarkable higher TF concentration than a pure withered leaf fermentation, with or without added tannase. The TF concentration was closely related to the catechin concentration in the fermentation and the ratio of non-ester/ester catechins. The use of tannase in the tea manufacture increased the TF concentration 4.7-fold. The TF concentration of black tea beverage was increased by a relatively low amount of withered leaves (6 g), a low fermentation temperature (25 °C), the absence of buffer and pH control, an intermediate fermentation time (60 min) and a relatively low aeration rate (0.8–1.0 L/min). Further optimization studied unveiled the most promising conditions for tea processing: 6.16 g of withered leaves (in 400 mL fermentation broth), fermentation for 63 min, at an aeration rate of 0.82 L/min. Studies on the sensory profile and bioactivity of black teas produces under these optimized conditions are necessary.

## Declaration of Competing Interest

The authors declare that they have no known competing financial interests or personal relationships that could have appeared to influence the work reported in this paper.
