# Supplementary material for: Optimization of a tannase-assisted process for obtaining teas rich in theaflavins from Camelia sinensis leaves
Source: Food Chem X. 2022 Jan 3;13:100203. doi: 10.1016/j.fochx.2022.100203 (PMC9039937; doi:10.1016/j.fochx.2022.100203)
Supplement: Supplementary data 1 [file mmc1.docx]

**Optimization of a tannase-assisted process for obtaining teas rich in theaflavins from *Camelia sinensis* leaves**

Shuang Liang ^a, b^, Fang Wang ^a^, Jianxin Chen ^a^, Daniel Granato ^c^, Lijun Li ^d^,

Jun-Feng Yin ^a^, Yong-Quan Xu ^a, *^

^a^*Tea Research Institute Chinese Academy of Agricultural Sciences, Key Laboratory of Tea Biology and Resources Utilization, Ministry of Agriculture, 9 South Meiling Road, Hangzhou 310008, China*

^b^*Graduate School of Chinese Academy of Agricultural Sciences, Beijing 100081, China.*

*^c^Department of Biological Sciences, Faculty of Science and Engineering, University of Limerick, V94 T9PX Limerick, Ireland. E-mail:* [*daniel.granato@ul.ie*](mailto:daniel.granato@ul.ie)

*^d^College of Food and Biological Engineering, Jimei University, Xiamen 361021, China*

**Corresponding Authors**

^*^Yong-Quan Xu, Tel: +86-571-86650594. Fax: +86 571 86650056. Email: [yqx33@126.com](mailto:yqx33@126.com).


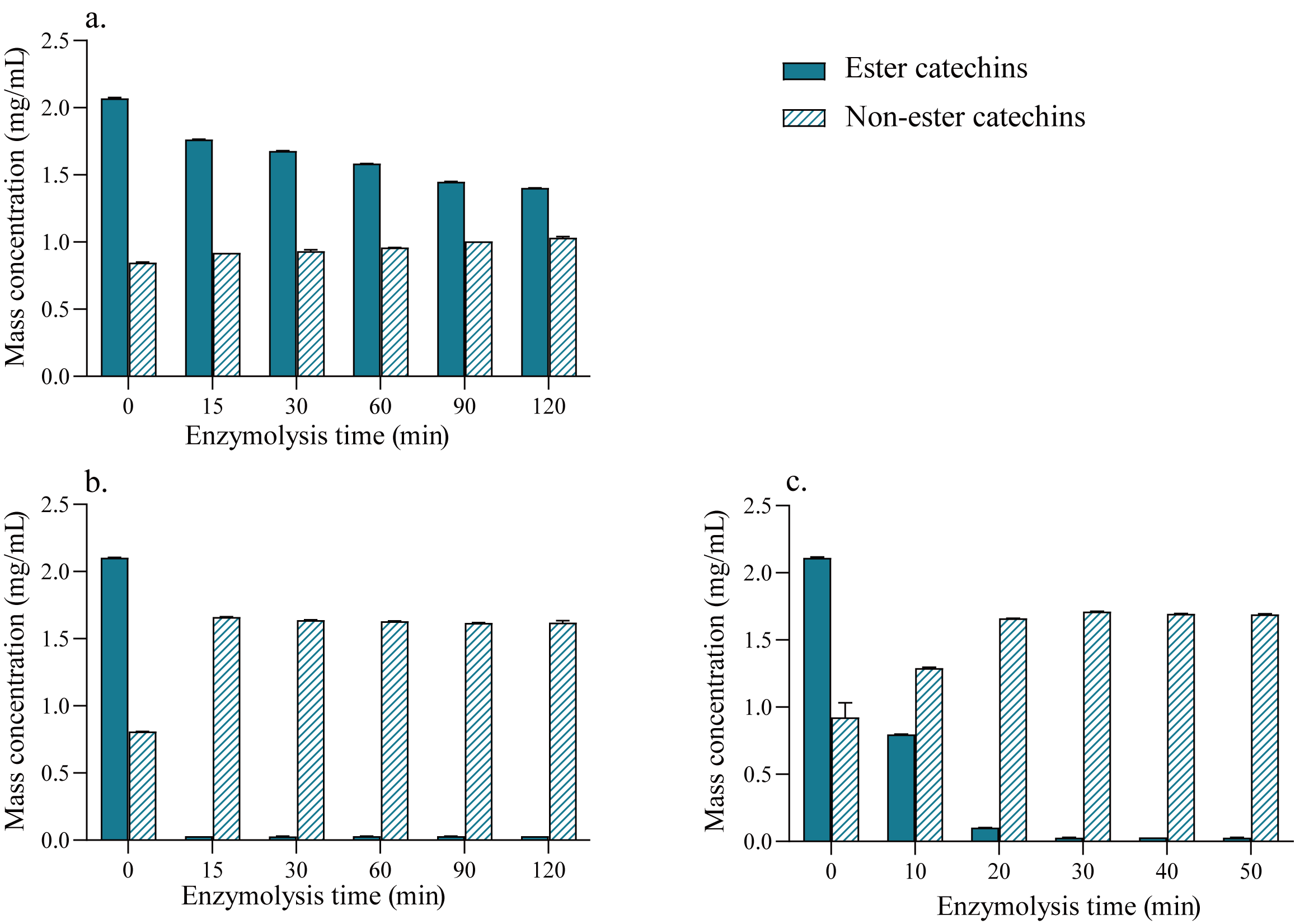


**Fig. S1. Effects of different additions of tannase to green tea extract on the time-courses of catechin content**

(a) 0.05% w/w tannase; (b) 0.25% tannase; (c) 0.1% tannase.

**
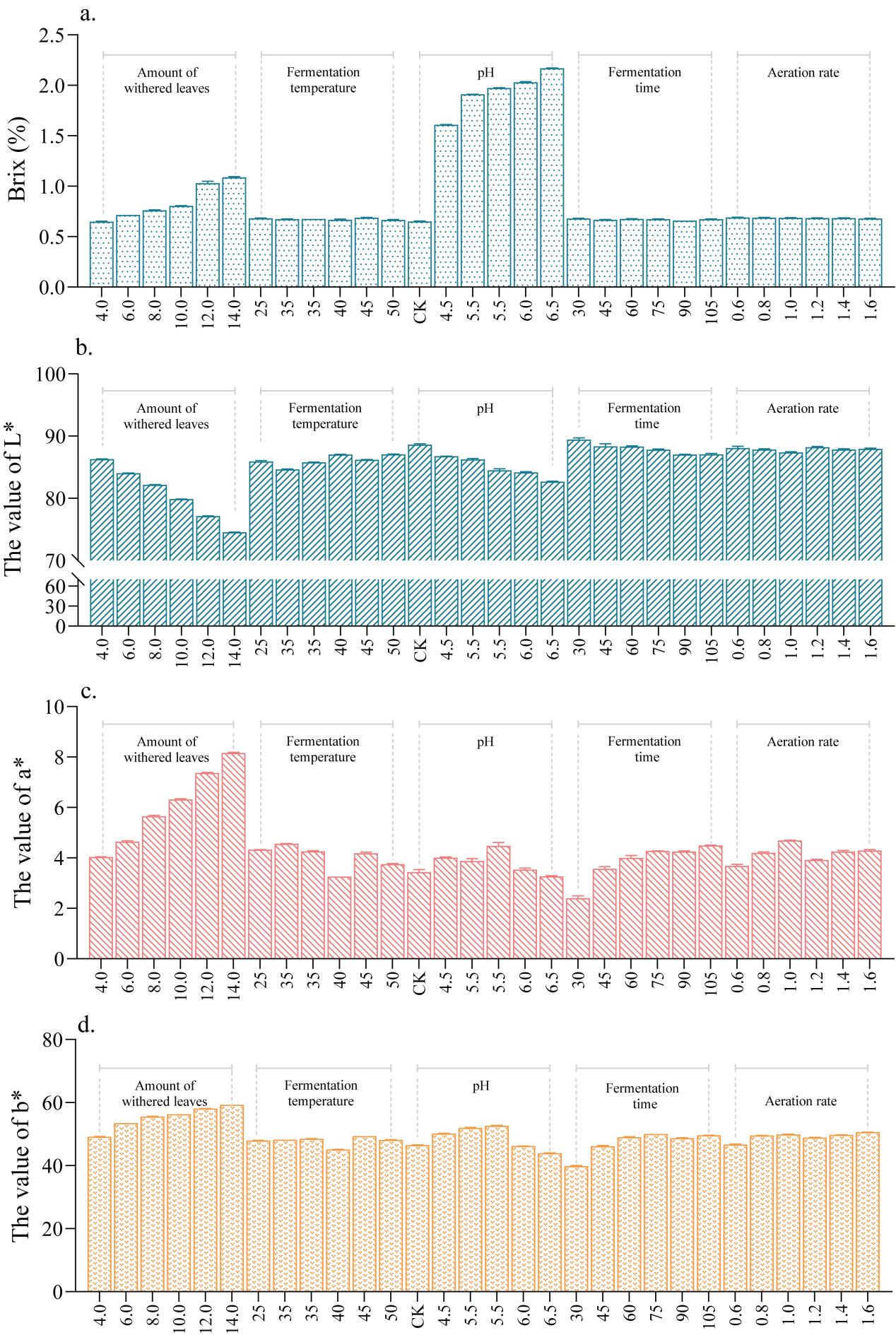
**

**Fig. S2. Effect of independent variables on the color of fermented tea juice in liquid-state fermentation**

(a) Brix, (b) *L** (Lightness; higher is whiter) value, (c) *a** (green/red; higher is redder) value, (d) *b** (blue/yellow; higher is yellower) value. The variables were: amount of withered leaves (g; in a 400 mL fermentation), fermentation temperature (^o^C), pH of fermentation liquid, fermentation time (min) and aeration rate (L/min). Error bars represent the average standard deviation from six independent experiments, carried out in triplicate.

**Table S1 Box-Behnken design with experimental results**

| Run | Independent variables coded and uncoded | | | Y^a^ |
| --- | --- | --- | --- | --- |
|  | A/g | B/min | C/(L/min) |  |
| 1 | -1(4) | 0(60) | 1(1.0) | 0.224 |
| 2 | 1(8) | 0(60) | 1(1.0) | 0.235 |
| 3 | 0(6) | -1(45) | 1(1.0) | 0.245 |
| 4 | 0(6) | 1(75) | 1(1.0) | 0.254 |
| 5 | 0(6) | 0(60) | 0(0.8) | 0.261 |
| 6 | -1(4) | 0(60) | -1(0.6) | 0.238 |
| 7 | -1(4) | 1(75) | 0(0.8) | 0.241 |
| 8 | -1(4) | -1(45) | 0(0.8) | 0.231 |
| 9 | 0(6) | 0(60) | 0(0.8) | 0.267 |
| 10 | 1(8) | -1(45) | 0(0.8) | 0.233 |
| 11 | 1(8) | 0(60) | -1(0.6) | 0.239 |
| 12 | 1(8) | 1(75) | 0(0.8) | 0.239 |
| 13 | 0(6) | -1(45) | -1(0.6) | 0.235 |
| 14 | 0(6) | 0(60) | 0(0.8) | 0.270 |
| 15 | 0(6) | 1(75) | -1(0.6) | 0.252 |
| 16 | 0(6) | 0(60) | 0(0.8) | 0.268 |
| 17 | 0(6) | 0(60) | 0(0.8) | 0.269 |

^a^, Y represents the mass concentration of TF (mg/mL)

**Table S2 Variance analysis of Box-Behnken experiments**

| Source | *F*-value | *P*-value | *R^2^* |
| --- | --- | --- | --- |
| Model | 62.14 | < 0.0001 | 0.9876 |
| A | 14.85 | 0.0063 |  |
| B | 20.41 | 0.0027 |  |
| C | 6.37 | 0.0395 |  |
| AB | 0.14 | 0.7182 |  |
| AC | 0.035 | 0.8563 |  |
| BC | 1.27 | 0.2967 |  |
| A^2^ | 329.19 | < 0.0001 |  |
| B^2^ | 82.47 | < 0.0001 |  |
| C^2^ | 59.78 | 0.0001 |  |
| Lack of fit | 3.13 | 0.1496 |  |
| *CV* |  | 2.12 |  |
| *R^2^_Adj_=0.9717* | *R^2^_Pred_=0.8555* | signal-to-noise ratio=16.531 | |
